# Comparative cytogenetics of nine populations of the *Rhinella* genus (Anura, Bufonidae) with a highlight on their conservative karyotype

**DOI:** 10.1590/1678-4685-GMB-2018-0139

**Published:** 2019-06-27

**Authors:** Daniel Pacheco Bruschi, Deborah Yasmim Sousa, Amanda Soares, Klélia Aparecida de Carvalho, Carmen Sílvia Busin, Natália Cristine Ficanha, Albertina Pimentel Lima, Gilda Vasconcellos Andrade, Shirlei Maria Recco-Pimentel

**Affiliations:** 1 Departamento de Genética, Setor de Ciências Biológicas, Universidade Federal do Paraná (UFPR), Curitiba, PR, Brazil; 2 Departamento de Biologia Estrutural e Funcional, Instituto de Biologia, Universidade Estadual de Campinas (UNICAMP), Campinas, SP, Brazil; 3 Departamento de Genética e Evolução, Microbiologia e Imunologia, Instituto de Biologia, Universidade Estadual de Campinas (UNICAMP), Campinas, SP, Brazil; 4 Instituto de Ciências Biológicas, Programa de Pós-Graduação em Ciências Ambientais (PPGCiAmb), Universidade de Passo Fundo (UPF), Passo Fundo, RS, Brazil; 5 Instituto Nacional de Pesquisas da Amazônia (INPA), 69060-001 Manaus, AM, Brazil; 6 Departamento de Biologia, Centro de Ciências Biológicas e da Saúde, Universidade Federal do Maranhão (UFMA), São Luís, MA, Brazil

**Keywords:** Bufonid toads, nucleolar organizing region, chromosomal evolution

## Abstract

The genus *Rhinella* is one of the most diverse groups of bufonid toads, currently composed by 93 valid species and naturally distributed throughout different Neotropical ecoregions. Here, we analyze nine Brazilian populations of toads representing species of the *Rhinella margaritifera* and *Rhinella marina* groups. These new data include the first description of the *R. hoogmoedi* and *R. proboscidae* karyotypes, as well as other taxonomically unresolved forms. Chromosomal analysis of the populations revealed pronounced chromosomal uniformity (2n=22), including the diploid number and chromosomal morphology. Three different NOR-bearing chromosomes were identified: in the subterminal region of pair 10q in *R. hoogmoedi*, *Rhinella* sp. 1 and *Rhinella* sp. 2, in subterminal region of 7p in *R. proboscidae* and *Rhinella* cf. *margaritifera* while in *R. henseli* and *R. icterica* was detected in interstitial region of 7p. Karyotypic uniformity of the genus permits the inference of interspecific chromosome homologies and evolutionary changes in the NOR-bearing chromosome may represent an informative character in species group level. The review of the cytogenetic data of the *Rhinella* species together with the new karyotypes reported here contributes to the understanding of the chromosomal evolution of these toads, which karyotypes are highly conserved despite the ample distribution of many forms.

Cytogenetic data provide a powerful tool for the evaluation of the taxonomic (Cuevas, 2008; Fávero *et al.*, 2011; Funk *et al.*, 2012) and evolutionary relationships (Veiga-Menoncello *et al.*, 2014) among anuran species. The understanding of chromosomal characters helps to identify synapomorphies (Cunningham and Cherry, 2004; Targueta *et al.*, 2012; Suárez *et al.*, 2013; Ferro *et al.*, 2018) and homoplasies (Cardozo *et al.*, 2011), and when combined with molecular phylogenetic inferences, these can contribute to the understanding of the role of chromosomal rearrangements in the diversification of a lineage (Veiga-Menoncello *et al.*, 2014).

Evolutionary analysis of anuran cytogenetics has provided important additional insights for phylogenetic inferences (Lourenço *et al.*, 2015; Targueta *et al.*, 2018). Recent shifts in analytical approaches have allowed more systematic evaluations,that have traced evolutionary changes in the chromosomal complement (*e.g.*, Lourenço *et al.*, 2008), both within and among anuran groups. However, the lack of cytogenetic data for many groups remains a limiting factor, especially if taking into account the considerable taxonomic richness of the order Anura, which has at least 7,058 species (*sensu* Frost, 2019). An interesting example is the genus *Rhinella*, a cosmopolitan group currently composed of 92 valid species (*sensu* Frost, 2019), although there is considerable evidence of a number of cryptic species and undescribed taxa (Fouquet *et al.*, 2007a). Most of the species of this genus are arranged in species groups (*R. crucifer, R. festae, R. granulosa, R. margaritifera, R. marina, R. spinulosa*, and *R. veraguensis*), but some taxa have not been assigned to any existing group (Chaparro *et al.*, 2007; Moravec *et al.*, 2014).

Cytogenetic analyses of the genus *Rhinella* revealed a pronounced chromosomal uniformity, including the diploid number and chromosomal morphology, and some species, such as *Rhinella icterica*, *Rhinella jimi*, and *Rhinella schneideri*, cannot even be distinguished by their C-banding or the distribution of their NOR (Kasahara *et al.*, 1996; Amaro-Ghilardi *et al.*, 2007). However, alternative NOR-bearing chromosomes have been identified in other species of the genus (Silva, 2010; Baraquet *et al.*, 2011). The present study is based on a compilation of the available chromosomal data for *Rhinella*, combined with karyotypes obtained from nine Brazilian populations of toads representing species of the *Rhinella margaritifera* and *Rhinella marina* groups. These new data include the first description of the karyotypes of two species of the *R. margaritifera* group (*Rhinella hoogmoedi* and *Rhinella proboscidae*), as well as other taxonomically unresolved forms.

The specimens and their respective collecting localities are listed in Table 1. The collection of specimens was authorized by SISBIO/Chico Mendes Institute for the Conservation of Biodiversity, through license number 20266-1. Voucher specimens were deposited in the Zoology Museum (ZUEC) “Prof. Dr. Adão José Cardoso” at Campinas State University (UNICAMP) in Campinas, SP, Brazil. The chromosomal samples were prepared from suspensions of intestinal epithelial cells, following King and Rofe (1976) and Schmid (1978). The chromosomes were stained with 10% Giemsa or submitted to the Ag-NOR technique (Howell and Black, 1980). The chromosomes were ranked and classified according to the criteria of Green and Session (1991). In addition to these primary data, the Web of Science (Institute of Scientific Information, Thomson Scientific) bibliographic database was searched for all the published cytogenetic data available on the genus *Rhinella*.

**Table 1:** Number of *Rhinella* specimens analyzed and their localities in Brazil.

Species	Number of specimens analyzed	Collection locality	Geographical coordinates
*R. hoogmoedi*	2 M + 3 F	Bertioga, SP	23º48’23.15”S; 46º03’32.23”O
*R. proboscidae*	2 M + 1 F	Reserva Florestal Adolpho Ducke, Manaus, AM	2º57’48.04”S; 59º55’22.20”O
*Rhinella* sp. 1	2 M + 1 F	Bacabeira, MA	2º56’28.32”S; 44º21’41.35”O
*Rhinella* sp. 2	5 M	Parque Viruá, RR	1º17’26.82”N; 61º09’09.20”O
*Rhinella* cf. *margaritiffera*	5 M + 1 F	Laranjal do Jari, AP	1º05’39.49”N; 53º13’04.98”O
*R. henseli*	2 M + 5 F	FLONA,sPasso Fundo, RS	28º18’59.10"S; 52º11’26.42’O
*R. henseli*	3 M + 3 F	Sertão, RS	28º02’33.46"S; 52º12’58.56"O
*R. icterica*	3 M + 2 F	FLONA, Passo Fundo, RS	28º18’59.10"S; 52º11’26.42’O
*R. icterica*	8 M	Sertão, RS	28º02’33.46"S;52º12’58.56"O

M: male; F: female

Chromosomal analysis of the nine *Rhinella* populations revealed conservative karyotype features, beginning with the diploid number (2n = 22), which was consistent across all species (see Table 2; Figure 1). All karyotypes consist of six metacentric (pairs 1–3, 5, 8 and 9; Fig. 1) and five submetacentric pairs (pairs 4, 6, 7, 10 and 11; Fig.1). While relatively few *Rhinella* species have been analyzed cytogenetically (Table 2), the available data are remarkable for their uniformity, with only metacentric and submetacentric pairs being found in any species. Small differences in the number of metacentric and submetacentric pairs are found in some studies (Amaro-Ghilardi *et al.*, 2008; Baraquet *et al.*, 2011), however, it seems likely that this has been due to the application of different criteria for the classification of the chromosomes, rather than any real variation among species in their karyotypes.

**Table 2:** Detailed cytogenetic data available for species of the *Rhinella* genus.

Species	2n	FN	NOR-bearing chromosome	Reference
*R. margaritifera* group				
*R. hoogmoedi*	22	44	St 10(p)	Present study
*R. margaritifera*	22	44	St 10(q)	Baldissera *et al.* (1999)
*Rhinella* sp. 1	22	44	St 10(q)	Present study
*Rhinella* sp. 2	22	44	St 10(q)	Present study
*Rhinella cf.margaritifera*	22	44	St 7(p)	Present study
*R. proboscidea*	22	44	St 7(p)	Present study
*R. crucifer group*				
*R. crucifer*	22	44	St 7(p)	Kasahara *et al*., (1996) Silva (2010)
*R. ornata*	22	44	St 7(p)	Silva (2010)
*R. pombali*	22	44	St 7(p)	Silva (2010)
*R. marina group*				
*R. arenarum*	22	44	Int 7 (p)	Baldissera *et al.* (1999) Baraqueti *et al.* (2011)
*R. icterica*	22	44	Int 7 (p)	Kasahara *et al.* (1996) Baldissera *et al.* (1999) Azevedo *et al.* (2003) Present study
*R. marina*	22	44	Int 7 (p)	Baldissera *et al,*(1999)
*R. schneideri*	22	44	Int 7 (p)	Kasahara *et al,*(1996) Azevedo *et al.* (2003) Amaro-Ghilardi *et al.* (2008) Baraquet *et al.,*(2011)
*R. rubescens*	22	44	Int 7 (p)	Amaro-Ghilardi *et al.,*(2008)
*R. jimi*	22	44	Int 7 (p)	Amaro-Ghilardi *et al.* (2008)
*R. achavali*	22	44	Int 7 (p)	Kolenc *et al.* (2013)
*R. henseli*	22	44	Int 7 (p)	Present study
*R. granulosa group*				
*R. granulosa*	22	44	Ter 5(q)	Baldissera *et al.* (1999)
*R. pygmaea*	22	44	Ter 5(q)	Baldissera *et al.* (1999)
*R. fernandezae*	22	44	n.e.	Baraquet *et al.* (2011)
*R. spinulosa group*	n.e.	n.e.	n.e.	n.e.
*R. veraguensis group*	n.e.	n.e.	n.e.	n.e.
*R. festae group*	n.e.	n.e.	n.e.	n.e.
*R. achalensis* (not assigned to any group)	22	44	Int10(p)*	Baraquet *et al.* (2011)

q = long arm; p = short arm; St= subtelocentric; int =interstitial position; per = pericentromeric position; ter = terminal position in the chromosome; n.e. = not examined; *only secondary constriction information.

**Figure 1:**
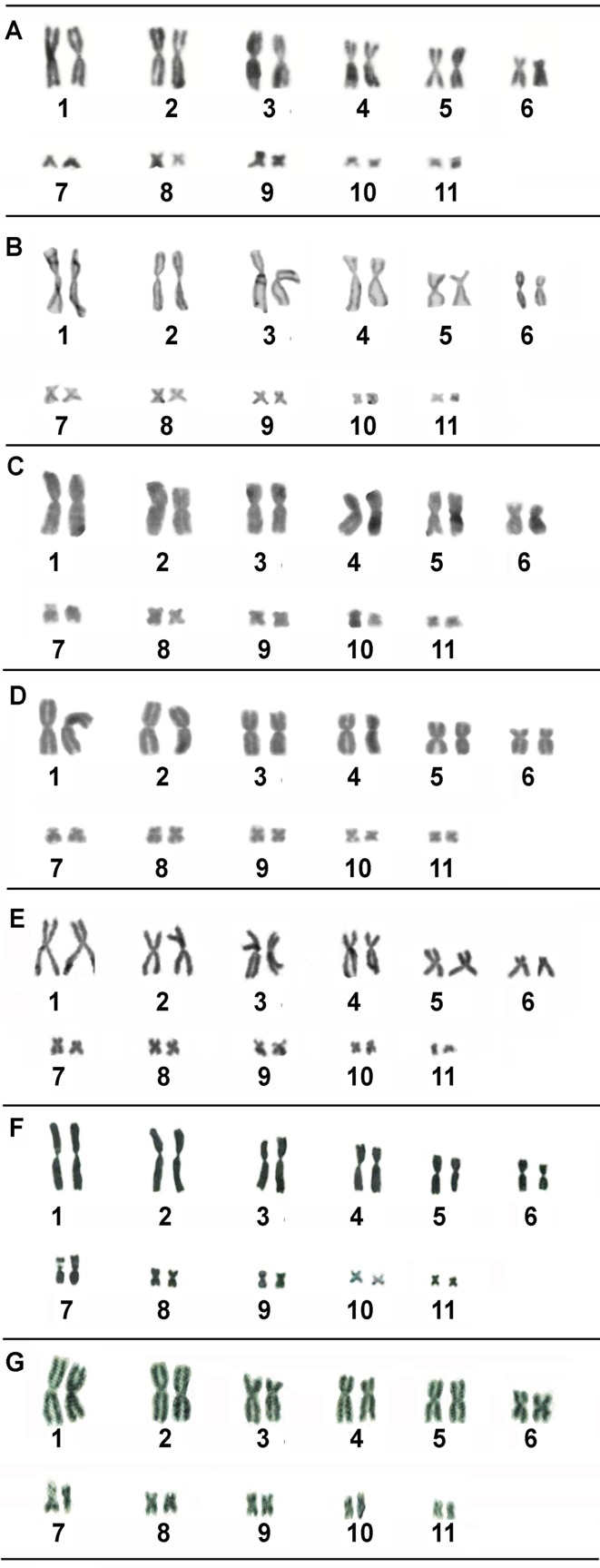
Karyotypes of five species of the *Rhinella margaritifera* (A-E) and *Rhinella marina* (F-G) groups from Brazil, based on Giemsa staining: (A) *R. hoogmoedi*; (B) *R. proboscidae*; (C) *Rhinella* sp. 1; (D) *Rhinella* sp. 2 and (E) *Rhinella* cf. *margaritifera*; (F) *R. ictarica* from Sertão, RS; (G) *R. henseli,* from Sertão, RS.

Three different NOR-bearing chromosomes were identified in the present study. In *R. hoogmoedi* (Bertioga, SP), *Rhinella* sp. 1 (Bacabeira, MA), and *Rhinella* sp. 2 (Parque Viruá, RR), the silver impregnation method detected a NOR site in the subterminal region of the long arm of pair 10, while in *R. proboscidae* (Reserva Ducke, AM) and *Rhinella* cf. *margaritifera* (Laranjal do Jari*,* AP) the NOR was located in the subterminal region of the short arm of the homologs of pair 7 (Figure 2). In two species of the *R. marina* group, *Rhinella henseli*, and *Rhinella icterica*, from both Passo Fundo and Sertão (RS), NOR-bearing chromosomes were detected in the interstitial region of the short arm of the homologs of pair 7, in both sampled populations (Figure 2).

**Figure 2:**
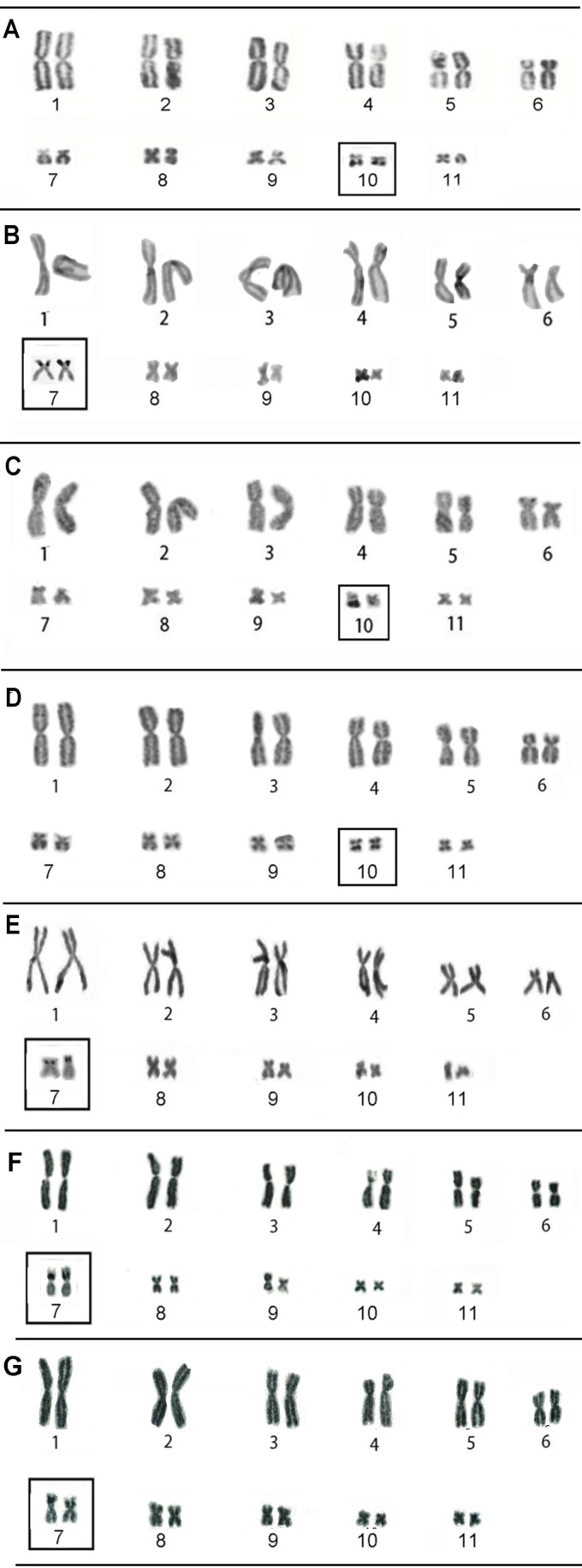
Karyotypes of five species of the *Rhinella margaritifera* (A-E) and *Rhinella marina* (F-G) groups from Brazil stained by the Ag-NOR method: (A) *R. hoogmoedi*; (B) *R. proboscidae*; (C) *Rhinella* sp. 1; (D) *Rhinella* sp. 2, and (E) *Rhinella* cf. *margaritifera*; (F) *R. ictarica,* from Sertão, RS; (G) *R. henseli,* from Sertão, RS*.* The boxes indicate the NOR-bearing chromosomes.

A similar degree of uniformity has been observed in inter-population cytogenetic studies of other *Rhinella* species. For example, considerable karyological uniformity has been found in the *R. icterica* populations from three sites in the Brazilian state of São Paulo (Kasahara *et al.*, 1996; Baldissera *et al.*, 1999; Azevedo *et al.*, 2003) and in the two populations from Rio Grande do Sul (present study). The *cururu* toad, *R. icterica*, occurs in southern Brazil, ranging from Rio Grande do Sul in the South to Bahia in the Northeast, as well as Minas Gerais and Goiás, eastern Paraguay, and extending westward to Missiones in Argentina. It is considered a cosmopolitan species, occurring in different habitats and altitudes, including within the Atlantic Forest biome. The cytogenetic data available on the populations of *R. schneideri*, another species distributed widely in South America, indicate a similar pattern of uniformity (Kasahara *et al.*, 1996; Azevedo *et al.*, 2003; Amaro-Ghilardi *et al.*, 2007; Baraquet *et al.*, 2011).

The overview of the data available for the different *Rhinella* species (Table 2) indicates that the position of the NOR site is relatively stable within phenotypic groups in the genus. The *Rhinella* species groups are determined based on phenotypic criteria (Frost, 2018), and while the monophyly of the *Rhinella granulosa* group has received support from molecular phylogenetic analyses (Pereyra *et al.*, 2016), this has not been confirmed in the others. Karyotype data are available for four of these species groups (Table 2). In all the species of the *Rhinella crucifer* group, the NOR is found in the subtelocentric region of pair 7p, whereas in the *R. marina* group, it is found in an interstitial region of the 7p, and in the *R. granulosa* group, it is located in the terminal region of the homologs of pair 5q. In the *R. margaritifera* group, by contrast, the NOR site is found in the subterminal region of the homologs of pair 10q or the interstitial region of pair 7p (Table 2).

As the karyotypic uniformity of the genus permits the inference of interspecific chromosome homologies, it seems reasonable to conclude that the evolutionary changes in the NOR-bearing chromosome may represent a putative synapomorphy in each species group. However, the confirmation of this hypothesis would require the analysis of a much larger dataset, for a more reliable evaluation of potential synapomorphies. If confirmed, the variation in the characteristics of the NOR-bearing chromosomes would provide valuable insights for the understanding of the phylogenetic relationships among the *Rhinella* species groups. In the *R. granulosa* group, for example, Pereyra *et al.* (2016) identified NORs in the homologs of pair 5 as an additional synapomorphy in this group. A similar scenario can be inferred for both the *R. crucifer* and *R. marina* groups, but not the *R. margaritifera* group, given the variation already observed in the NOR-bearing chromosomes of the different species of this group.

The *R. margaritifera* group is currently composed of 19 species (Vaz-Silva *et al.*, 2015), with the NOR being found in the homologs of pair 10 (long arm) in *R. hoogmoedi* (present study), and in *Rhinella* sp. 1 from Maranhão and *Rhinella* sp. 2 from Roraima. Baldissera *et al.* (1999) described the karyotype in specimens of the *R. margaritifera* group from Tucuruí, in the Brazilian state of Pará. In the present study, three populations (*Rhinella* sp. 1, *Rhinella* sp. 2, and *Rhinella* cf. *margaritifera*) were assigned to the *R. margaritifera* group based on morphological and biogeographical criteria, although it was not possible to determine the taxonomic status of these populations based on their karyotypes. Despite their morphological similarities, the *Rhinella* cf. *margaritifera* specimens from Laranjal do Jari can be distinguished from all the other populations assigned to the *R. margaritifera* group based on the NOR-bearing chromosome and by phenotypic features (Lima - personal observation), which may indicate the presence of a novel taxonomic entity, which requires further investigation.

The taxonomic status of the species of the *R. margaritifera* group remains uncertain, and phylogenetic inferences indicate the existence of a number of cryptic lineages, and a possible species complex within this group (Fouquet *et al.*, 2007a). This reinforces the need for a thorough taxonomic review of the arrangement of the *R. margaritifera* group in the Amazon region. Molecular approaches have been effectively applied to the recognition and description of many new *Rhinella* species (Fouquet *et al.*, 2007b; Moravec *et al.*, 2014), and it would almost certainly provide important insights into the delimitation of the species within the *R. margaritifera* group.

The species of the *Rhinella margaritifera* group are distributed in northern South America and the Central America forest domain, except for *R. hoogmoedi* (Caramaschi and Niemeyer, 2003), which inhabits the Atlantic Forest biome, and *R. scitulla*, *R. ocellata, R. sebbeni*, and *R. paraguayensis*, which occur in the Brazilian Cerrado savanna (Ávila *et al.*, 2010; Vaz-Silva *et al.*, 2015). However, the forest-dwelling species tend to present high levels of individual variation in morphological features, which limits the usefulness of these attributes for the discrimination of species. Santos *et al.* (2015) identified populations from western Ecuador and Panama, frequently assigned to *R. margaritifera* species, as *R. alata*, which has helped to resolve the confusing zoogeography of the *R. margaritifera* complex. However, the status of the populations of the *R. margaritifera* group from the east of the Andes remains unresolved, and the phylogenetic inferences of Fouquet *et al.* (2007a) indicated the potential existence of at least five distinct taxa identified as *R. margaritifera* in Brazil and French Guiana.

The *R. proboscidae* karyotype is described here for the first time, and it presents a NOR on the homologs of pair 7, a condition different from that of the other species of the *R. margaritifera* group. While the taxonomy and arrangement of the species in this group are complex (Fouquet *et al.*, 2007a), it has been diagnosed by the presence of an expansion of the posterior ramus of the pterygoid (Pramuk, 2006), a putative morphological synapomorphy that supports the *R. margaritifera* group. However, this putative phenotypic synapomorphy has not been formally tested. *Rhinella proboscidae* occurs along the Amazon River between Peru and Manaus, in Brazil, the locality sampled in the present study. The morphological characters of these populations support their inclusion in the *R. margaritifera* group. In contrast with the other species groups, however, in which the NOR-bearing chromosome represents a putative synapomorphy, two distinct scenarios are equally possible for the *R. margaritifera* group: (1) the NOR on pair 10 is a chromosomal synapomorphy in this group and the NOR on pair 7 of *R. proboscidae* represent a character reversion; or (2) the retention of an ancestral polymorphism. Unfortunately, the lack of a complete phylogenetic reconstruction that includes representatives of all the *Rhinella* groups hampers more conclusive inferences on the chromosomal evolution of this genus. In the specific case of the *R. margaritifera* group, a more systematic analysis of monophyly based on the investigation of specific molecular markers would likely provide decisive insights into the evolution of this group.

The conservative arrangement of the NOR-bearing chromosomes in the different *Rhinella* groups highlights the potential contribution of cytogenetic data for the identification of diagnostic synapomorphies in species groups or clades. A similar approach has been applied successfully in other amphibian groups (see Grant *et al.*, 2017; Targueta *et al.*, 2018). For example, comparative cytogenetics and the allocation of chromosomal characters (morphology and NOR sites) in a phylogenetic tree inferred from molecular markers allowed Cardozo *et al.* (2011) to identify three potential synapomorphies in the genus *Ololygon* (*Scinax catharinae* clade – Duellman *et al.*, 2016). Lourenço *et al.* (2015) also identified the interstitial C-band in chromosome pair 5 as a synapomorphy of the *Physalaemus cuvieri* species group.

Another relevant feature of *Rhinella* genus is the high frequency of hybridization and introgression events (Azevedo *et al.*, 2003; Narvaes and Rodrigues 2009; Sequeira *et al.*, 2011; Guerra *et al.*, 2011; Pereyra *et al.*, 2015) in areas of sympatry, mainly in the *Rhinella marina* group. As chromosomal features may represent important pre- or post-zygotic barriers to reproduction, groups with a uniform karyotype, such as those found in *Rhinella*, may reflect the relaxation of any isolation mechanism, which would further contribute to the high frequency of hybridization events observed in this genus. For example, Azevedo *et al.* (2003) identified an intermediate form between *Rhinella icterica* and *Rhinella schneideri* in a sympatric zone, based on the banding patterns of seroproteins analyzed by electrophoresis, even though the intermediate form presented no modification of the karyotype in comparison with the parental species.

Overall, it is hoped that this review of the cytogenetic data available for the *Rhinella* species, together with the new karyotypes reported here, will contribute to the understanding of the mechanisms of evolutionary changes that led to the diversification of these toads. Despite the ample distribution of many forms, karyotypes are highly conserved in most cases.
